# Pentameric Architecture of the SARS-CoV‑2 Envelope Protein Revealed by SEC-MALS, Cryo-EM, and Molecular Dynamics

**DOI:** 10.1021/acs.jpcb.5c08730

**Published:** 2026-03-12

**Authors:** Zi-Wen Weng, Danny Farhat, Jyh-Yeuan Lee, Shang-Te Danny Hsu

**Affiliations:** † Institute of Biological Chemistry, 38017 Academia Sinica, 128 Academia Road, Sec. 2, Nankang, Taipei 11529, Taiwan; ‡ Institute of Biochemical Sciences, National Taiwan University, 1 Roosevelt Road, Sec. 4, Daan, Taipei 10617, Taiwan; § Department of Biochemistry, Microbiology, and Immunology, Faculty of Medicine, 6363 University of Ottawa, Ottawa, Ontario K1H 8M5, Canada; ∥ International Institute for Sustainability with Knotted Chiral Meta Matter (WPI-SKCM^2^), Hiroshima University, 1-3-1 Kagamiyama, Higashi-Hiroshima, Hiroshima 739-8526, Japan

## Abstract

The SARS-CoV-2 envelope (E) protein is a small viroporin that drives viral assembly, budding, and host interactions, yet its structural organization has remained elusive. Earlier nuclear magnetic resonance spectroscopy studies hint at oligomerization without direct evidence, and the construct lacks the flexible C-terminal region. To bridge this gap, we synthesized the full-length E protein to investigate its oligomeric state. Using size-exclusion chromatography coupled with multiangle light scattering, we demonstrated that the E protein assembles as a stable pentamer in solution. We then reconstituted the E protein into membrane scaffold protein nanodiscs to mimic the lipid bilayer environment for structural analyses by negative-stain electron microscopy and cryo-electron microscopy, which revealed pentamer-like features. Molecular dynamics simulations of the E protein in a nanodisc and a membrane bilayer setting further corroborated the structural flexibility of the C-terminal domain. Collectively, these data present direct evidence that the SARS-CoV-2 E protein assembles as a pentamer in both solution and membrane-mimetic environments. Our results provide a structural foundation for future investigations into the E protein’s roles in ion channel activity, membrane remodeling, and virus–host interactions.

## Graphical abstract

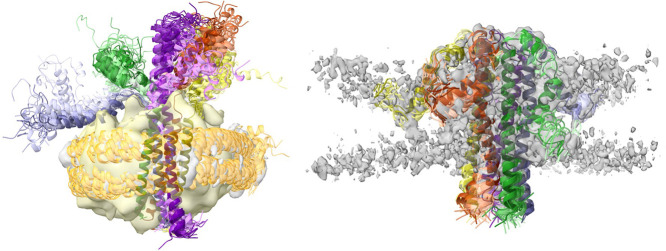

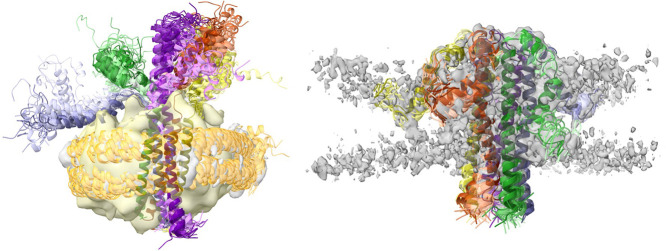

## Introduction

The envelope (E) protein of SARS-CoV-2 is a small viroporin of 75 amino acids in length. It serves multiple important functions, including contributing to viral assembly and budding, affecting host cell survival, activating immune responses, and disrupting cell polarity. Its N-terminal transmembrane domain (TMD) is proposed to form a pentameric assembly^1,2^ that serves as a calcium ion channel;^3^ its C-terminal domain interacts with the human Zona Occludens-1 (ZO1), which mediates tight junctions^4,5^ (Figure 1). The E and membrane (M) proteins are essential for inducing membrane curvature and envelope formation during viral assembly.^6,7^ Its absence results in aberrant viral morphology and compromised virus production, underscoring its pivotal role in the viral life cycle.^8^ Comparative studies have revealed that coronavirus E proteins can be categorized into three groups based on their hydropathy plots,^9^ with variations in the number and location of transmembrane domains and conserved cysteine regions. These cysteine residues – C40, C43, and C44 in SARS-CoV-2 E protein – are susceptible to palmitoylation, which may enhance hydrophobicity, thereby facilitating membrane insertion or lipid bilayer interactions^10^ (Figure 1). Biochemical evidence from studies on murine coronavirus (MCV)^11,12^ and infectious bronchitis virus (IBV)^13^ supports the role of cysteine palmitoylation in the E-M protein interactions and efficient viral assembly.^11,14,15^

**Figure 1:**
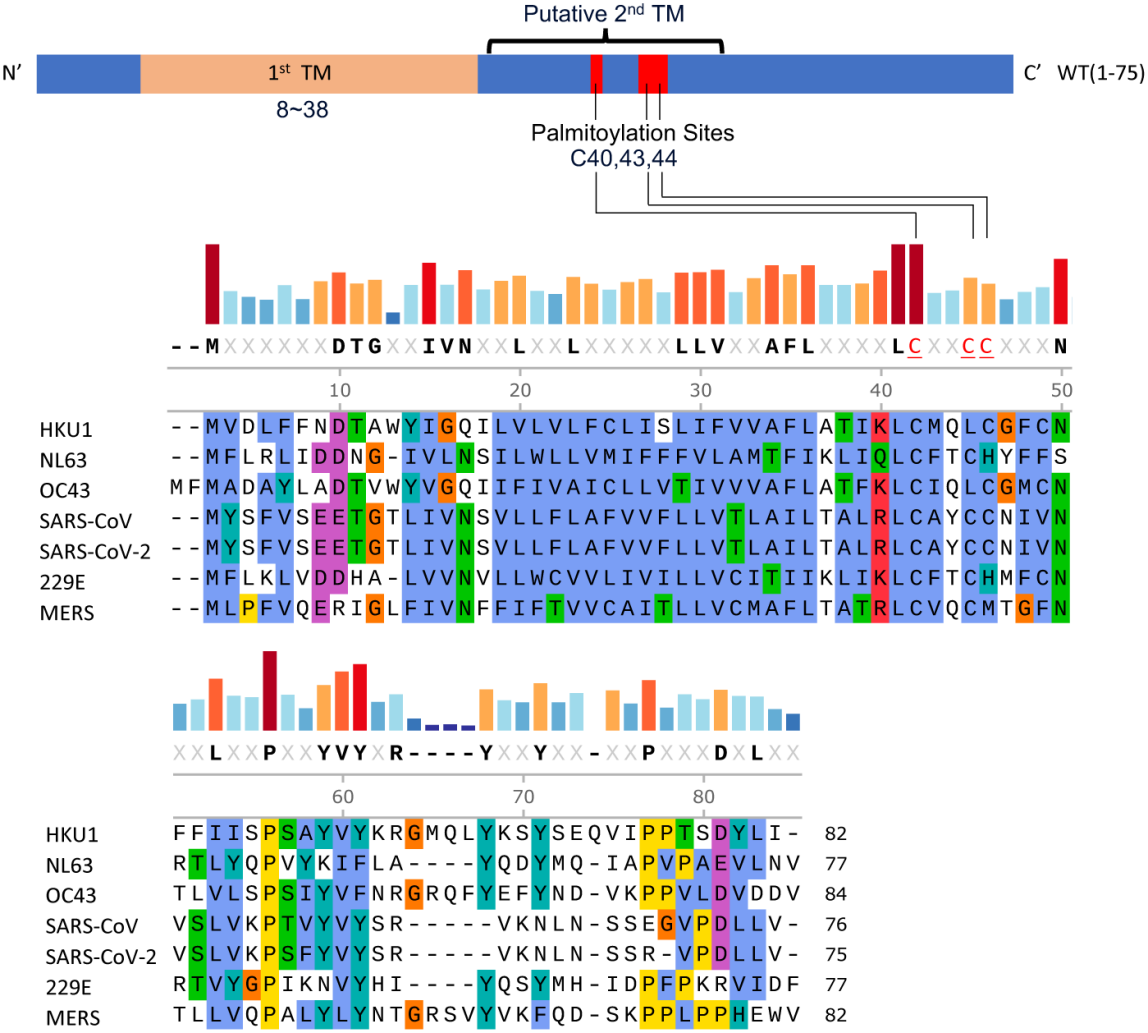
Multiple sequence alignment (MSA) and domain definition of the E proteins of SARS-CoV-2 and other human coronaviruses. The multiple sequence alignment was done by ClustalW.^16^ The three conserved cysteines – C40, C43, and C44 in SARS-CoV-2 – that are putatively palmitoylated are highlighted red in the consensus sequence above the MSA.

The E protein ensures proper viral assembly by regulating the localization and conformation of the spike (S) protein, facilitating viral targeting and maturation.^17^ As a viroporin, the E protein forms ion channels that disturb host cell ion homeostasis, reduce cell viability, and trigger inflammatory responses.^18^ Despite its importance, the comprehensive structure–function relationship remains elusive, particularly regarding the C-terminal region, the functional contribution of palmitoylation, and the exact oligomeric state. Indeed, the native architecture of the SARS-CoV-2 E protein has been a subject of ongoing debate. While many functional models and solid-state nuclear magnetic resonance (ssNMR) studies strongly advocate for a pentameric channel assembly,^1,6^ recent investigations have revealed remarkable structural plasticity. For instance, recent ssNMR studies demonstrated that truncated constructs encompassing both the transmembrane and N-terminal ectodomains can adopt dimeric states even within native-like lipid bilayers.^2^ Furthermore, earlier studies found that certain detergent environments can induce artificial mixtures of lower-order oligomers. These discrepancies highlight that the E protein’s assembly is acutely sensitive not only to its surrounding hydrophobic environment but also to the exact length of its sequence; using truncated constructs can fundamentally alter its oligomeric propensity. To unambiguously resolve this controversy, we chemically synthesized a full-length E protein with an N-terminal polyhistidine tag (87 amino acids) and reconstituted it in detergent micelles and membrane scaffold protein (MSP)-stabilized nanodiscs to characterize its oligomeric state using size-exclusion chromatography-coupled static multiangle light scattering (SEC-MALS). Furthermore, we used electron microscopy (EM) to visualize the oligomeric assembly of the E protein, followed by all-atom molecular dynamics (MD) simulations to investigate the dynamics of the E protein within a nanodisc and a membrane bilayer environment.

## Materials and Methods

### SARS-CoV-2 E Protein Synthesis, Purification, and Nanodisc Reconstitution

The full-length SARS-CoV-2 E protein (hereafter E protein) construct, which includes an N-terminal polyhistidine tag (the complete amino acid sequence is provided in Figure S1), was chemically synthesized using standard Fmoc solid-phase peptide synthesis (Fmoc-SPPS). The synthesis was performed on an automated microwave peptide synthesizer (Liberty Blue, CEM Corporation). Following the synthesis, the peptide was cleaved from the resin, and the side-chain protecting groups were concurrently removed using a cleavage cocktail consisting of trifluoroacetic acid (TFA), water, and triisopropylsilane (TIS) at a ratio of 95:2.5:2.5 (v/v/v). The crude protein sample was subsequently obtained through ether-mediated precipitation and dried under high vacuum. Then the synthetic product was further purified by fast protein liquid chromatography (FPLC; AKTA pure 25L, Cytiva, USA) using a size exclusion chromatography column (Superdex 200 increase 10/300, Cytiva, USA) to homogeneity, confirmed by matrix-assisted laser desorption/ionization time-of-flight mass spectrometry (MALDI-TOF MS; SCIEX 5800 TOF/TOF Tandem Mass Spectrometer, SCIEX, USA). The organic solvent was removed by lyophilization, resuspended, and solubilized in absolute methanol (Sigma-Aldrich, USA). Methanol was removed by vaporization on a heat block (Thermomixer, Eppendorf, Germany) at 25̊C, 800 rpm, followed by the use of streams of nitrogen gas. The E protein was solubilized in Buffer A (50 mM Tris-HCl (pH 6.2), 150 mM NaCl), supplemented with 2 (w/v) % n-dodecyl-β-D-maltoside (DDM; Anatrace, USA). The mixture was incubated at 4̊C for 3–16 h, followed by filtration through a 0.22 μm filter and centrifuged at 10,000 × *g* for 2 min (FA-45-24-11 rotor with Eppendorf 5424/5424R centrifuge; Eppendorf, Germany). The DDM-solubilized E protein was further purified by FPLC (UPC10, Cytiva, USA) using an analytical SEC column (Superdex 200 increase 10/300; Cytiva, USA) in Buffer B (50 mM Tris-HCl (pH 6.2), 150 mM NaCl, and 0.02 (w/v) % DDM) as the mobile phase. The E protein-containing fractions were pooled and concentrated using a centrifugal concentrator (Amicon Ultra 30 kDa MWCO, USA) at 3000 g (A-4–62 rotor with Centrifuge 5810 R; Eppendorf, Germany). The protein concentration was determined by measuring the absorbance at 280 nm using a NanoPhotometer N60 (IMPLEN, Germany) with a theoretical extinction coefficient of 6085 M^–1^ cm^–1^.

### Far-UV Circular Dichroism Spectroscopy

The purified E protein was buffer-exchanged from Buffer B to Buffer C (50 mM sodium phosphate (NaPi), pH 6.2, 0.02 (w/v)% DDM) and concentrated to a final volume of 50 μL. A 0.5 mL Amicon Ultra device with a 3k MWCO concentrator was equilibrated in Buffer C for the second buffer exchange and concentration step. Iterative dilution and concentration steps were performed to remove impurities and residual chemicals. First, 450 μL of Buffer C was added to the concentrated sample to achieve a 10-fold dilution, followed by centrifugation at 4 °C, 13,000g for 20–30 min to reduce the sample volume to approximately 100 μL. Following this, three additional washing cycles were performed. In each cycle, 400 μL of the same buffer was added (5X dilution) and centrifuged under the same conditions, ultimately achieving a final sample volume of approximately 100 μL. Far-UV circular dichroism (CD) spectroscopy was used to assess the secondary structure of the purified E protein in Buffer C. Measurements were performed on a Jasco J-815 CD spectrometer over a wavelength range of 195–260 nm at 20 °C. A quartz cuvette (115-QS, Hellma, Germany) with a path length of 10 mm was used for all measurements. The protein sample concentration was adjusted to 0.150 mg/mL to achieve an optimal signal-to-noise ratio. The spectrometer parameters were configured as follows: the data integration time (D.I.T) was set to 1 s, the sample data pitch was 0.2 nm, the scan speed was 50 nm/min, and the bandwidth was 1.00 nm. The spectrum was obtained by averaging ten scans to minimize noise and improve data reliability. Baseline correction was performed using the spectrum of the corresponding buffer.

### E Protein Reconstruction in a Nanodisc

An engineered MSP, MSP1D1ΔH5,^19^ was used to reconstitute the E protein into a nanodisc. MSP1D1ΔH5 was transformed into *E. coli* BL21 cells, grown at 37 °C in LB medium supplemented with 50 μg/mL kanamycin overnight. The culture was then expanded in 1 L of medium at 37 °C. Overexpression of MSP1D1ΔH5 was induced by the addition of 1 mM isopropyl β-D-1-thiogalactopyranoside (IPTG) when the cell culture density reached an optical density at 600 nm (OD_600_) of 0.7, and further culturing at 16 °C for 15 h. Bacterial cells were harvested by centrifugation using the JLA-8.1 rotor at 6,000 rpm for 30 min, and the cell pellet was collected after removing the supernatant. For MSP1D1ΔH5 protein purification, the frozen cell pellet (from 2 L bacterial culture) was resuspended in 50 mL lysis buffer containing 50 mM Tris-HCl (pH 8.0), 500 mM NaCl, 1% Triton X-100, 1 mM EDTA, supplemented with one protease inhibitor tablet (cOmplete, Roche, Germany), 1 mg lysozyme, 5 mM MgCl_2_, and 1 mg DNase. The resuspended cells were passed through a 25-G needle (Terumo Corp., Japan) twice and incubated on ice for 1 h. Cell lysis was achieved by sonication (10 min, 10 Amp, 10 s work/10 s pause) followed by additional lysis using a Nanolyzer N2 (GoGene Corporation, Taiwan). The lysate was centrifuged using the JLA8.1 rotor at 18,000 rpm for 30 min at 4 °C, and the supernatant was collected and filtered through a 0.2 μm filter.

The His-tagged MSP1D1ΔH5 protein was purified using Ni-NTA affinity chromatography. The cOmplete His-Tag purification resin (Roche, Germany) 1 mL resin per 1 L of cell culture) was equilibrated with 50 mM Tris-HCl (pH 8.0), 500 mM NaCl, and 1% Triton X-100. The filtered supernatant was applied to the column three times to ensure complete binding. The column was washed sequentially with 5 column volumes (CV) of Tris buffer with 1% Triton X-100, 10 CV of Tris buffer without detergent, 10 CV of Tris buffer with 1% Triton X-100, 10 CV of Tris buffer with 50 mM cholate, 10 CV of Tris buffer without detergent, and 10 CV of Tris buffer with 20 mM imidazole. The MSP1D1ΔH5 protein was eluted with 7 mL of Tris buffer containing 500 mM imidazole. The protein concentration was determined using NanoPhotometer N60 (IMPLEN, Germany). An aliquot of the final product was subjected to sodium dodecyl sulfate-polyacrylamide electrophoresis (SDS-PAGE) analysis to confirm purity.

Purified MSP1D1ΔH5 was used to reconstitute the synthetic full-length E protein into a nanodisc, together with 1-palmitoyl-2-oleoyl-*sn*-glycero-3-phosphocholine (POPC), 1-palmitoyl-2-oleoyl-*sn*-glycero-3-phosphoethanolamine (POPE), and 1-palmitoyl-2-oleoyl-*sn*-glycero-3-phosphoserine (POPS). To prepare the nanodisc, the E protein, MSP1D1ΔH5, lipid mixture, and DDM were mixed in an Eppendorf microcentrifuge tube at molar ratios of 1:2:60:20:20:400 for E protein:MSP1D1ΔH5:POPC:POPE:POPS:DDM with a final protein concentration of 50 μM. The mixture was prepared in Buffer D (50 mM Tris-HCl (pH 7.4) and 150 mM NaCl) and incubated for 1 h at 4 °C. 200 mg/500 μL of Amberlite XAD2 (10357, Merck, USA) was added to the solution, followed by incubation at 4 °C for 16 h to remove DDM. After incubation, the biobeads were removed by puncturing the bottom of the Eppendorf tube with a heated needle, followed by centrifugation at 2000g for 2 min. The sample was then filtered using a 0.22 μm filter by centrifugation at 10,000 g for 3 min. The filtered sample was purified by FPLC using a Superdex 200 increase 10/300 (Cytiva, USA) SEC column and Buffer D to isolate the E protein-containing MSP nanodisc. The nanodisc was further concentrated to 1 mg/mL, aliquoted, flash-frozen in liquid nitrogen, and stored at −20 °C until further use.

### Size-Exclusion Chromatography-Coupled Multiangle Light Scattering (SEC-MALS)

The SEC-MALS analysis was carried out using an FPLC system (UPC10, Cytiva, USA) coupled to a quasi-elastic light scattering (QELS; Wyatt Technology, USA) detector with 18 detection angles and an in-line Optilab rEX differential refractive index (RI) detector (DAWN; Wyatt Technology, USA) as described previously.^20,21^ The E protein nanodisc was separated by a Superdex 200 10/300 increase column (Cytiva, USA) in Buffer D. The SEC-MALS data were processed using ASTRA v6 (Wyatt Technology, USA) using the extinction coefficients at 280 nm of 0.7610 mL mg^–1^ cm^–1^ (0.6108 for the E protein and 0.946 for the MSP1D1ΔH5), and the d*n*/d*c* values of 0.185 and 0.145 for amino acids and lipids, respectively, as inputs to deconvolute the stoichiometry to the E protein, MSP, and lipids in the nanodisc as described previously.^22–24^

### Negative-Stain Electron Microscopy (NSEM)

The E-MSP nanodisc complex was concentrated to 50 μg/mL for NSEM analysis. Four microliters of the stock solution were applied onto 300-mesh Quantifoil R1.2/1.3 holey carbon grids, which were glow-discharged (PELCO easiGlow 91000, Ted Pella Inc., USA) at 25 mA for 30 s to remove static charge before sample application to the carbon side. After 1 min of incubation at room temperature, the excess liquid was removed with filter paper. Subsequently, 4 μL of 2 (w/v) % uranium formate (UF) was added to the sample for 1 min. The excess stain was removed with filter paper. NSEM data were collected using an FEI Tecnai G2 F20 TWIN transmission electron microscope (TEM; FEI, The Netherlands) operated at 200 kV and equipped with a Gatan 4K × 4K CCD camera. Images were acquired at 62,000× magnification, corresponding to a calibrated pixel size of 1.326 Å/pixel, with an electron dose of approximately 40 e^–^/Å^2^ and a defocus range of −1.5 to −3.0 μm to optimize contrast.

### Cryogenic Electron Microscopy (Cryo-EM)

The E-MSP complex was concentrated to 1.0 mg/mL in a buffer containing 50 mM Tris-HCl (pH 7.4) and 150 mM NaCl. Quantifoil R1.2/1.3 300 mesh grids were glow-discharged for 20 s at 25 mA using a glow discharger (PELCO easiGlow 91000, Ted Pella Inc., USA). Aliquots of 4 μL of the purified protein were applied to the grids, blotted for 3 s under 100% humidity, 4 °C, and vitrified by plunge-freezing into liquid ethane using a vitrification robot (FEI Vitrobot Mark IV, ThermoFisher Scientific, USA).

Cryo-EM data were collected on a 300 kV Titan Krios TEM (ThermoFisher Scientific, USA) equipped with a Gatan K3 direct electron detector (Gatan, USA). Images were acquired in super-resolution mode at a nominal magnification of 120k×, corresponding to a calibrated pixel size of 0.55 Å/pixel. A total dose of 1.0 e^–^/Å^2^ was fractionated over 45 frames, with an exposure time of 0.022 s/frame. The defocus range was set between −1.2 and −1.8 μm to optimize contrast.

Motion correction and dose-weighting were performed using MotionCor2.^25^ Contrast transfer function (CTF) parameters were estimated using CTFFIND4.^26^ Particles were first picked using manual picking tools to build a template, followed by template picking tools, and extracted with a box size of 384 pixels. Two-dimensional (2D) classification was performed to remove poor-quality particles, and the remaining particles were subjected to ab initio model generation or initial 3D reconstruction using cryoSPARC.^27^

### Molecular Dynamics (MD) Simulation

To generate a full-length model for the E protein, we assembled a construct using previously reported structures^6,28^ and modeled the missing residues by AlphaFold2.^29^ Briefly, the N-terminal transmembrane domain, residues 8–38 of the pentameric SARS-CoV-2 E protein structure, determined by ssNMR spectroscopy (PDB ID: 7K3G)^6^ was joined to the C-terminal region of the SARS-CoV E protein structure determined by solution state NMR spectroscopy (PDB ID: 5X29)^28^ using Coot.^30^ The missing N- and C-terminal residues were modeled using AlphaFold2. Charmm-GUI^31^ was used to prepare initial models for all simulations. Default protonation states were kept, and the protein was inserted into either a nanodisc or a membrane following the same lipid ratio as the experimental nanodisc reconstruction, a 3:1:1 ratio of POPC:POPE:POPS. The system was charge-neutralized with 0.15 M NaCl using VMD^32^ and padded with 14 Å of water (an explicit solve model of TIP3P was used^33^ from the extremities of the protein–membrane system, resulting in a tetragonal box size of 132.1, 146.1, and 125.4 Å, containing 118526 atoms for the entire system. In the case of the E-MSP1D1ΔH5 simulation, our generated full-length model was placed into an MSP1D1ΔH5 nanodisc with the same 3:1:1 lipid ratio of POPC:POPE:POPS. This system was solvated with a padding of 14 Å of explicit water molecules with 0.15 M NaCl using VMD.^32^ All-atom MD simulations were carried out using NAMD3 ^34^ with CHARMM36/CHARMM36m force fields^35^ and TI3P water models.^33^ Water models were constrained through the SETTLE algorithm,^36^ and other bonds involving hydrogens were constrained using SHAKE/RATTLE^37,38^ algorithms. Hydrogen mass repartitioning^39^ allowed a 4 fs time-step for more efficient computing. The particle mesh Ewald method^40^ was used to calculate long-range electrostatics with a grid density of 1/Å. Short-range nonbonded interactions had a cutoff of 12 Å with a switch distance of 10 Å. Langevin dynamics and Langevin piston algorithm^41^ with dampening of 1/ps maintained the pressure at 1 atm and constant temperature at 310 K with periodic boundary conditions. All simulations followed a standard 3-step equilibration minimization protocol. The first step involved relaxing the lipid tails for 10 ns, followed by a second step that relaxed the membrane, water, and ions for another 10 ns. The third relaxation phase included the protein side chains, lasting for 20 ns, totaling 40 ns of relaxation time. Triplicate 120 ns production runs were conducted, and an analysis was performed using VMD and ChimeraX.^42^

## Results

### Oligomeric State of SARS-CoV-2 E Protein by SEC-MALS

To determine the oligomeric state of the chemically synthesized E protein reconstituted in DDM detergent micelles, we performed SEC-MALS to measure the molecular mass (MM) of the E protein as part of the micellar assembly. The SEC elution peak of the E protein was monodispersed, and the corresponding MM estimate was 54.34 ± 0.26 kDa (Figure 2A). The theoretical MM of the synthetic E protein monomer is 9.96 kDa (Figure S2). Therefore, the SEC-MALS analysis confirmed that the E protein in DDM micelles existed as a pentamer in solution. SDS-PAGE analysis of the main elution peak showed a single band at the expected molecular weight of the monomeric E protein (Figure S3).

**Figure 2:**
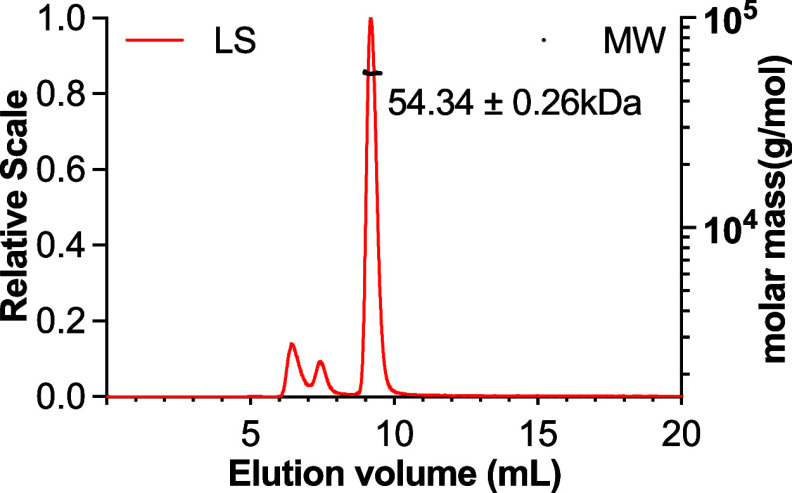
SEC-MALS analysis of SARS-CoV-2 E protein in DDM detergent micelles. The light scattering profile is shown in red, whereas the MM distribution is shown in black with the estimated values labeled alongside.

### Secondary Structure Analysis by Far-UV Circular Dichroism (CD) Spectroscopy

We used far-UV CD spectroscopy to assess the secondary structure composition of the DDM-solubilized SARS-CoV-2 E protein (Figure 3). The spectral deconvolution showed that the DDM-solubilized E protein contained 54.9% α-helical structure, 10.2% turns, and 3% β-stranded structure (Figure S2), consistent with the previously described secondary structural features of SARS-CoV E protein (PDB ID: 5X29) that shares a sequence homology of 94.7% (Figure 1).^43^

**Figure 3:**
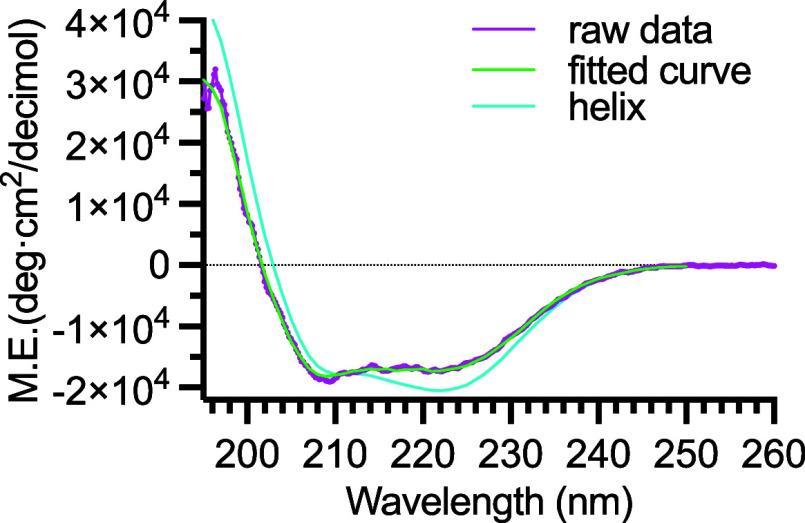
Far-UV CD spectrum of SARS-CoV-2 E protein reconstituted in DDM micelles. The E protein was buffered in Buffer C containing 0.02 (w/v) % DDM. The raw CD spectrum in magenta was superimposed with the spectral deconvolution in green to dissect the secondary structural contents. The theoretical spectrum of a 100% helical structure is shown in a dotted orange line.

### SEC-MALS Analysis of the E Protein in MSP Nanodisc

The oligomeric state of the E protein in MSP nanodisc was investigated using SEC-MALS. The SEC profile was more complex than that of the DDM-solubilized E protein. Nonetheless, utilizing Protein Conjugate Analysis based on the in-line UV, dRI, and MALS signals, we determined the overall MM of the first elution peak (elution volume ca. 12.4 mL) to be 126.9 kDa. By applying the respective *dn/dc* and extinction coefficient values, the mass of the protein core and the lipid modifier were independently calculated to be 87.8 kDa and 39.2 kDa, respectively (Figure 4A). The measured protein MM is consistent with the expectation for the sum of pentameric E protein plus two copies of the MSP (Figure 4B). Furthermore, the measured lipid MM contribution of 39.2 kDa precisely reflects the mass of the lipid bilayer surrounding the pentamer within this specific complex, which corresponds to 52 POPC molecules (760.1 Da per POPC). The SEC-MALS analysis showed successful incorporation of the E protein pentamer into a native-like membrane environment, paving the way toward further structural and functional studies of the E protein in a membrane context, which is crucial for understanding its role in viral assembly and host-cell interaction.

**Figure 4:**
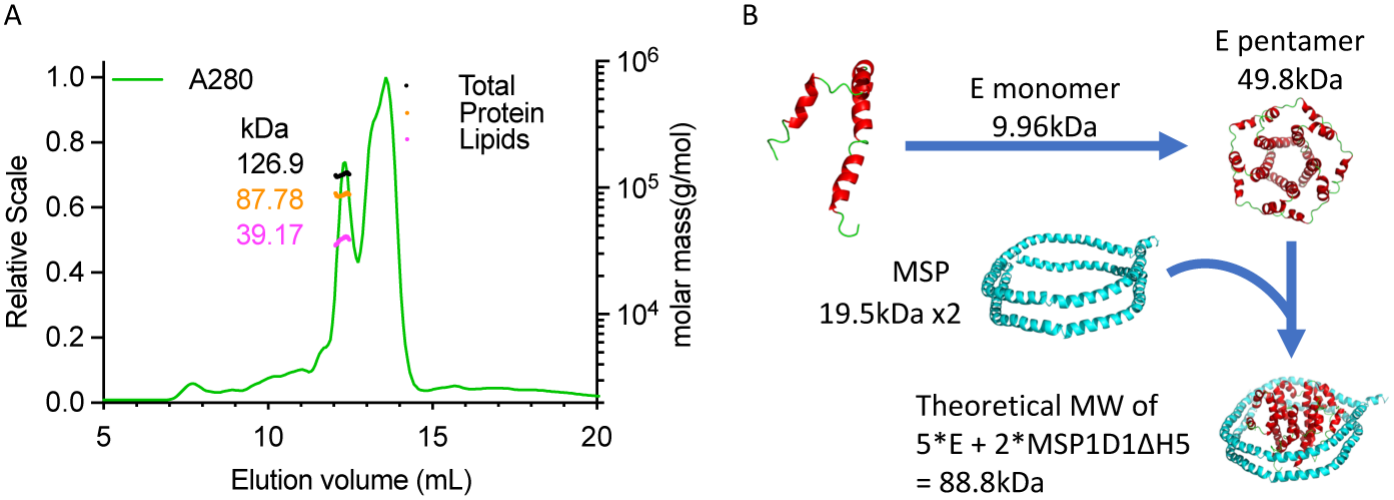
SEC-MALS confirmed the pentameric state of the E protein incorporated in the MSP nanodisc. (A) SEC-MALS analysis of E-MSP nanodisc complex. The SEC column was equilibrated with 50 mM Tris-HCl (pH 7.4), 150 mM NaCl, and the protein was dissolved in the same buffer. The chromatogram shows the LS, RI, and UV readings in red, blue, and green, respectively. The scale for the LS detector is shown on the left axis. The black, orange, and magenta lines indicate the calculated MM contributions of the overall assembly, the protein core (E protein + MSP), and the lipid modifier, respectively, determined via Protein Conjugate Analysis, with their estimated MMs shown on the right *Y*-axis. (B) Schematic diagram of the E-MSP nanodisc complex preparation.

### Structure and Oligomeric States of the E-MSP Nanodisc Complex by NSEM

The E protein reconstituted in MSP nanodiscs was further investigated by negative stain transmission electron microscopy (NSEM). After iterative particle image selections and extractions, the analysis of 66 micrographs yielded a data set of ca. 162,400 particle images (Figure 5). Well-defined disc-like particles were observed, corroborating the successful incorporation of the E protein into nanodiscs. Subsequent 2D classification revealed a homogeneous population of nanodiscs, supporting the monodispersity observed in SEC-MALS analysis. While the resolution of NSEM was insufficient to resolve the individual E proteins within the nanodisc, several 2D class averages showed pentamer-like density arrangements within the circular nanodisc. These structures, which likely correspond to five helices, are consistent with the pentameric organization of E protein determined by SEC-MALS. Some 2D classes showed a starfish-like density within the circular nanodisc profile, consistent with the expected arrangement of the five E protein C-terminal tails. While individual subunits are not resolvable at this resolution, the overall shape and size of the density are compatible with a pentameric assembly of E proteins embedded in the nanodisc. The NSEM results, in conjunction with the SEC-MALS data, provide compelling evidence for the successful reconstitution of E protein pentamers in nanodiscs.

**Figure 5:**
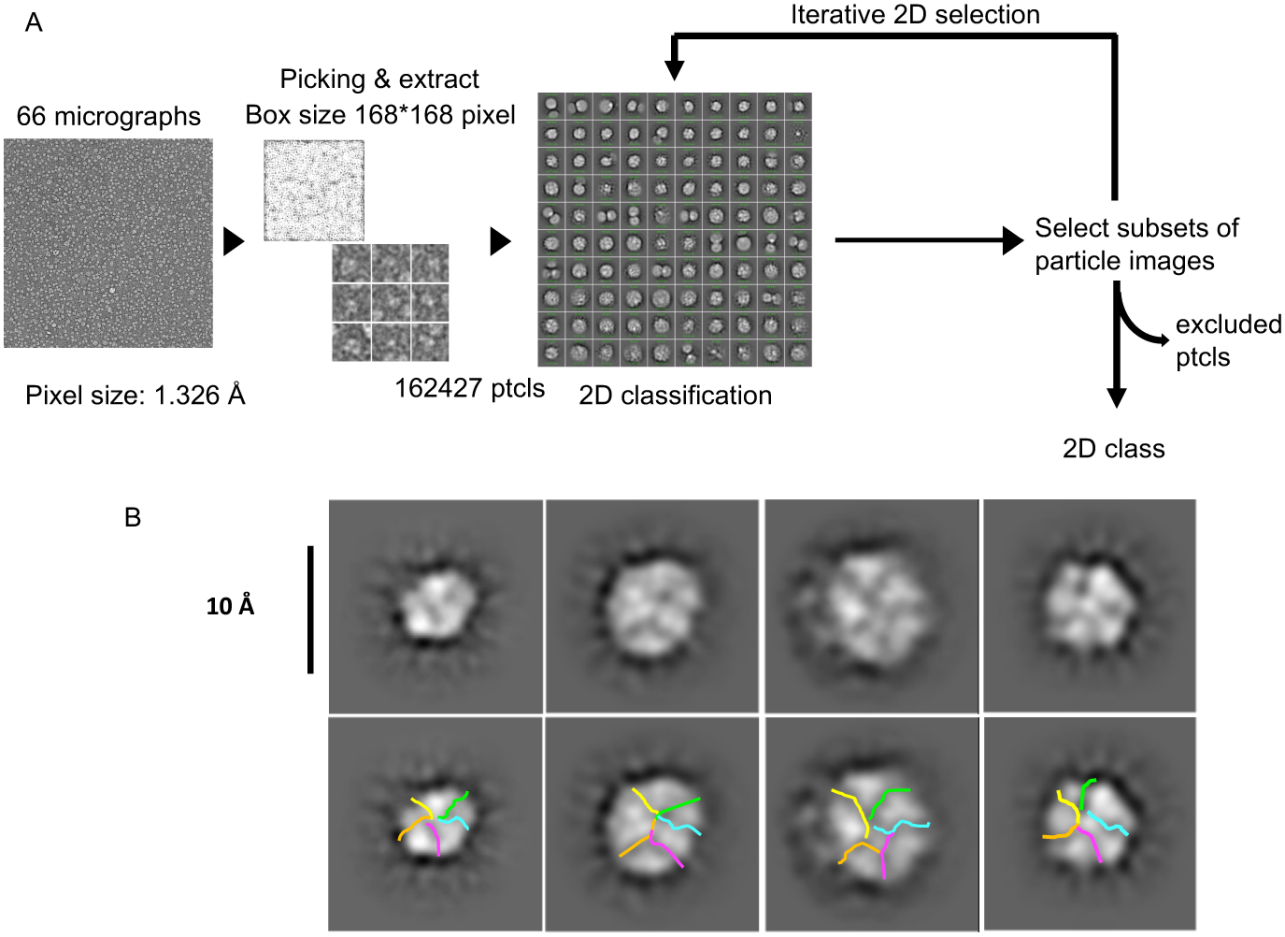
NSEM analysis of the E protein in nanodiscs. (A) Workflow of NSEM data processing. (B) Pentameric E protein in a nanodisc was observed in 2D classes.

### Structural Analysis of the E-MSP Nanodisc Complex by Cryogenic Electron Microscopy (Cryo-EM)

To obtain more detailed structural information, we performed single-particle cryo-EM on the E protein reconstituted in nanodiscs. Following 2D classification, most particle classes displayed a disc-like shape. The E protein appeared to be highly dynamic, making it intractable for reliable 2D classification for particle alignments. As such, the signal from the surrounding nanodiscs would dominate the averaged images, making it difficult to distinguish the relatively small and flexible E protein inside the nanodisc. After several attempts at 2D classification, the central density corresponding to the E protein remained blurry, resulting in a low-resolution 3D EM map where the pentameric arrangement could not be resolved (Figure 6).

**Figure 6:**
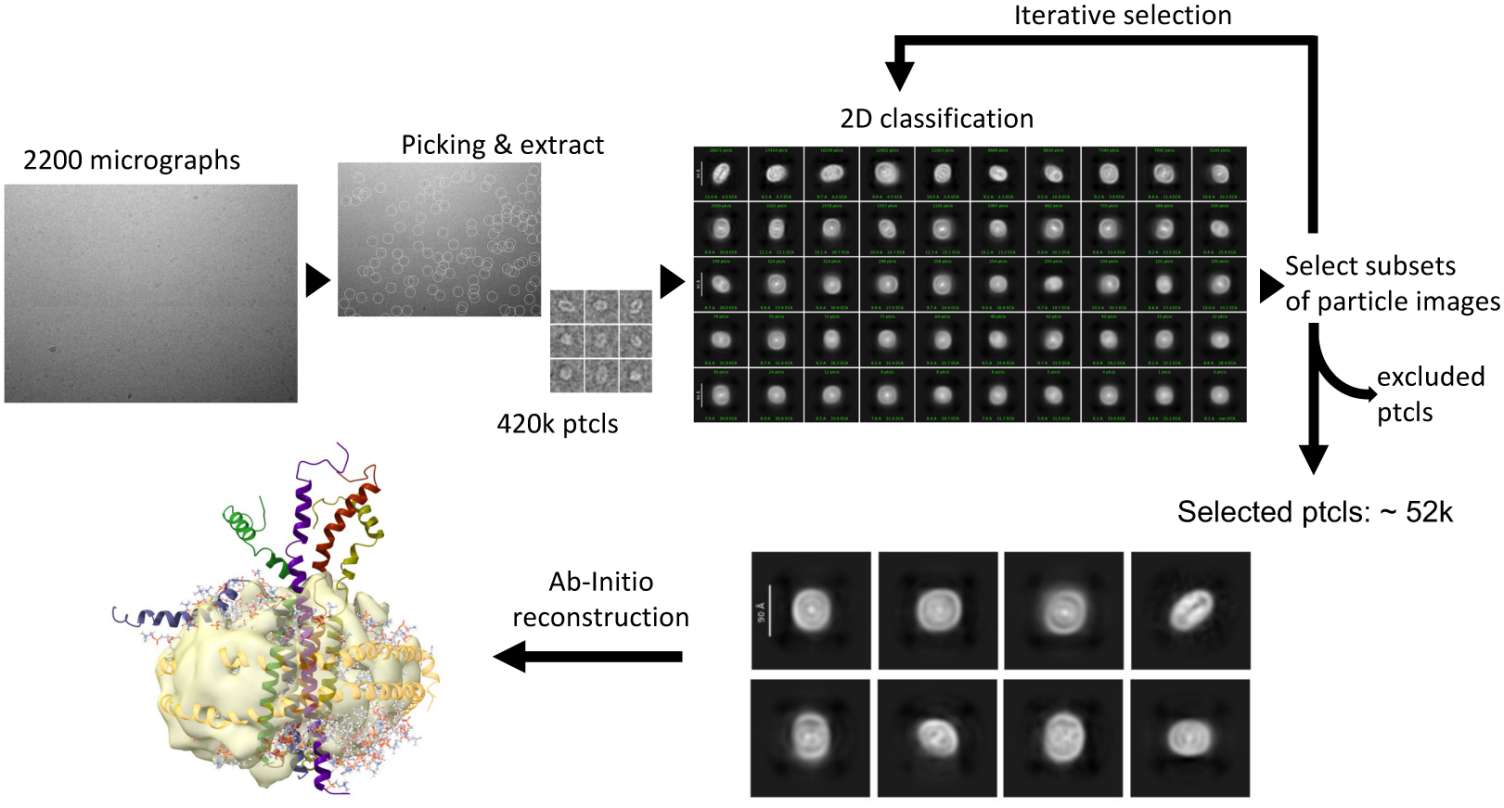
Cryo-EM analysis of SARS-CoV-2 E protein embedded in a nanodisc.

### Molecular Dynamics Simulation of the E Protein in a Nanodisc

To better understand the conformational flexibility and dynamics underlying these observations, we turned to MD simulations. We first built a full-length pentameric SARS-CoV-2 E protein model embedded in a nanodisc as the starting point for MD simulations. After various iterations, we settled on a model that contained the resolved transmembrane domain (PDB: 7K3G) and the C-terminal region of the SARS-CoV E protein structure (PDB: 5 × 29). AlphaFold2 was then used to fill the gaps, and the model was embedded in a nanodisc containing the same lipid ratio as used in our experiments, i.e., 3:1:1 for POPC:POPE:POPS.

Fitting the MD trajectories to the cryo-EM map confirmed the convergence of the MSP1DΔH5 nanodisc structure while highlighting significant protein dynamics. The simulation exhibited a global RMSD of ∼16 Å, driven primarily by the highly flexible C-terminus, which deviated by up to ∼25 Å. Snapshots of the protein were taken every 5 ns using VMD throughout the entire trajectory. These structures were aligned, and an artificial density map was generated using ChimeraX’s molmap function. This synthetic map correlates sufficiently (score: 0.47) with the experimental cryo-EM density. The synthetic map essentially resembles a disc with two perturbations above and below, validating that the protruding C-terminal tails account for the observed structural variability.

We carried out all-atom MD simulations for 120 ns, of which the first 20 ns (pre-equilibrated states) were excluded for further analyses. Superposition of the conformational ensemble of the E protein in MSP nanodisc from the trajectory of the MD simulation with the experimental cryo-EM map showed a good agreement in terms of the MSP nanodisc dimension and the central volume corresponding to the transmembrane domain of the E protein pentamer (Figure 7A and B). Quantitative analysis of the bending of the C-terminal helix with respect to the N-terminal TMD showed that the individual C-terminal tails of all five monomers (chains A-E) were very flexible throughout the last 100 ns trajectory (Figure 7C). These results strongly suggested that the poor resolution of our experimental map may partially or fully result from these highly flexible regions, which explains why cryo-EM 2D and 3D classifications failed to yield good image alignments and therefore a high-resolution EM map.

**Figure 7:**
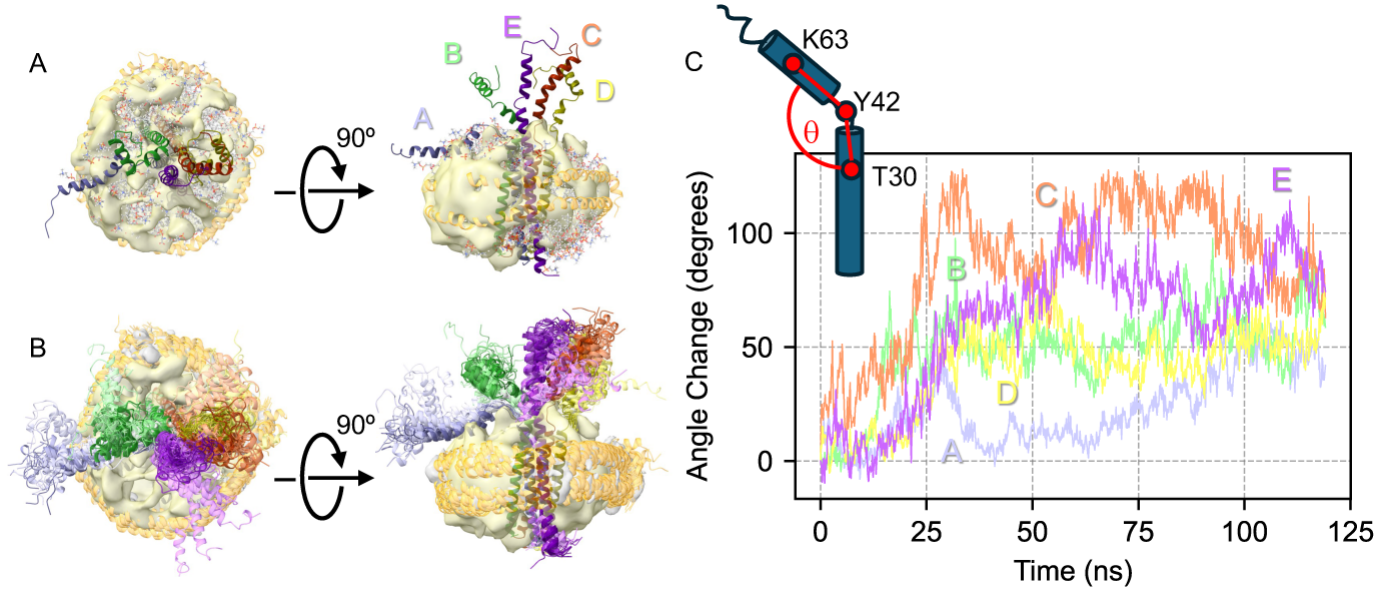
Comparison of cryo-EM map and MD simulations of the E protein pentamer in MSP1D1ΔH5 nanodisc. (A) Orthogonal views of the structure of the E protein pentamer in the MSP nanodisc at the end of the MD simulation. The E proteins are colored differently, and the MSPs are colored gold. (B) Orthogonal views of the conformational ensemble of the E protein pentamer in the MSP nanodisc. A conformational snapshot was taken along the trajectory every 5 ns, superimposed with the TMD inside the nanodisc. (C) Conformational flexibility of the C-terminal tail of the E protein is expressed as the interhelix angle θ defined schematically on the upper left corner. The interhelix angle as a function of the MD simulation time is plotted for the individual E protein monomers (chains A-E) with colors matching those in A and B.

### MD Simulations of the E Protein Pentamer in a Membrane Bilayer

To investigate the interplay between the E protein and the membrane environment, we carried out MD simulations of the E protein pentamer embedded in a membrane bilayer (Materials and Methods). In contrast to the high flexibility observed in the nanodisc (RMSD ∼ 16 Å), the E protein in the membrane bilayer was significantly more stable. When the protein backbone was restrained during the membrane simulation, we obtained a global RMSD of 8 Å, indicating the stability of the core structure in this environment. Unlike the MSP nanodisc simulation, the C-terminal tails of the E protein pentamer form stable contacts with the membrane bilayer surface, with partial insertion into the acyl chains of lipid molecules (Figure 8A). A synthetic EM map was generated by merging snapshots along the MD trajectory, which showed significant thinning of the membrane bilayer surrounding the E protein pentamer (Figure 8B).

**Figure 8:**
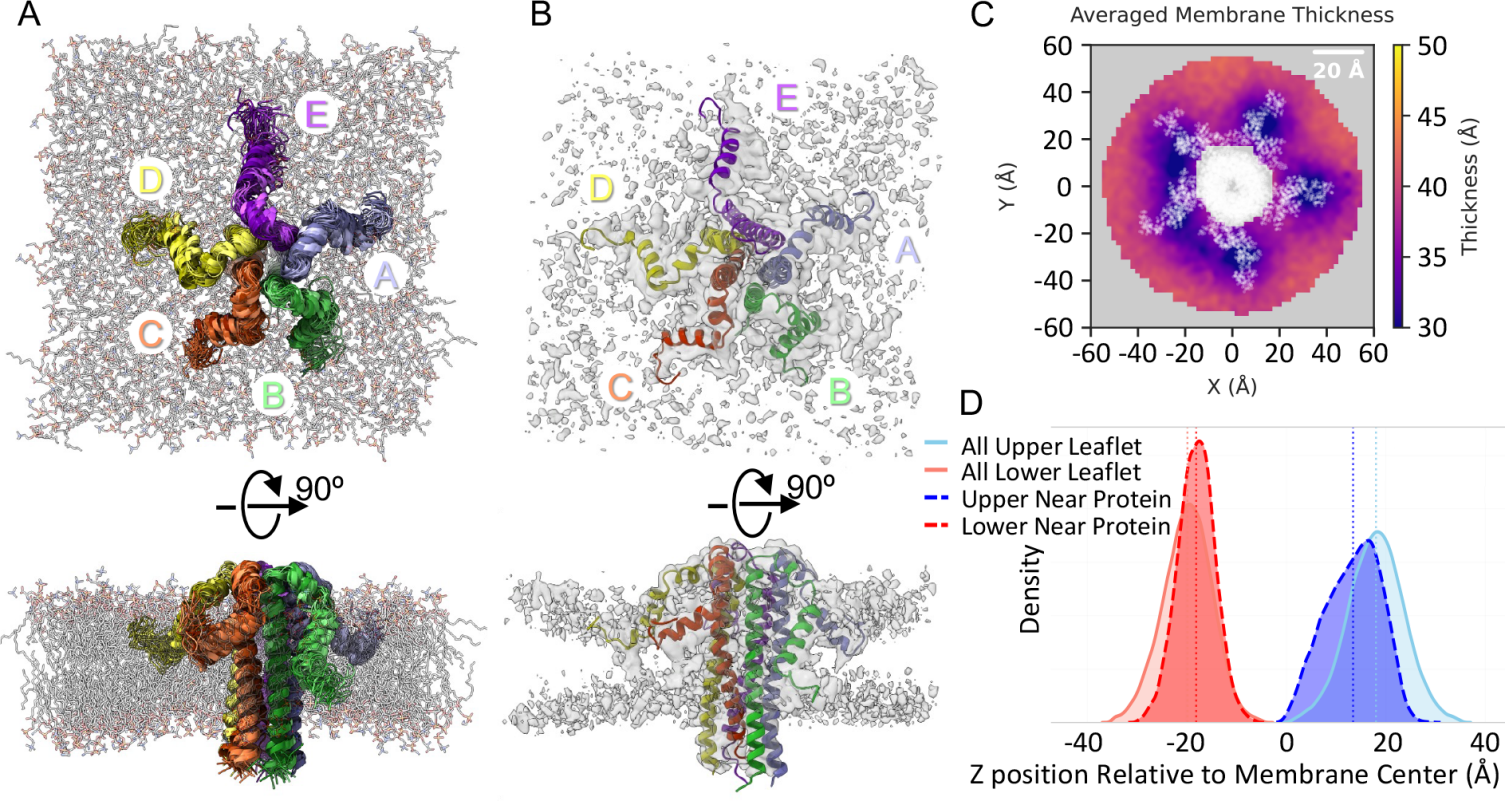
MD simulations of the E protein pentamer in a lipid bilayer. (A) Orthogonal views of the structure of the E protein pentamer in a lipid bilayer at the end of the MD simulation. The E proteins (chains A–E) are colored as in Figure 7. (B) Orthogonal views of the synthetic EM map of the MD trajectory (Materials and Methods) superimposed with the ensemble structures of the E protein pentamer. (C) Heatmap of average thickness over the MD trajectory. White dots represent the protein backbone average throughout simulations. (D) Density analysis of membrane thickness comparing near protein (10 Å from backbone) and overall membrane thickness, suggesting that within 10 Å of the protein, the distance between upper and lower leaflets is thinner compared to the same distance in the overall membrane.

To investigate the interplay between the E protein and the membrane bilayer, we monitored the spatial distribution of the actual thickness across the periodic box of the MD simulations (Figure 8C). The results revealed clear thinning near the E protein (within a radius of 10 Å), most prominent around the transmembrane helices. Quantitative analysis of the lipid density profiles (Figure 8D) elucidates the structural basis of the membrane thinning observed in Figure 8C. Comparisons between the bulk membrane (solid lines) and the lipids adjacent to the E protein (dashed lines) reveal a distinct shift in headgroup positions toward the membrane center. This thinning effect is asymmetric: the upper leaflet adjacent to the protein shifts inward by approximately 3 Å, whereas the lower leaflet exhibits a more pronounced inward shift of ∼5 Å. Furthermore, the density distribution of the lower leaflet near the protein (dashed red line) is significantly more dispersed than that of the upper leaflet, with a tail extending toward the membrane center, suggesting that the lower leaflet undergoes greater structural distortion and contributes more significantly to the overall membrane thinning.

## Discussion

Our study provides direct evidence confirming the pentameric assembly of the SARS-CoV-2 E protein in membrane-mimicking environments, addressing a critical gap in the structural characterization of this essential viroporin. By combining SEC-MALS, electron microscopy, and molecular dynamics simulations, we present a comprehensive characterization of the full-length E protein that extends beyond previous truncated constructs. Previous ssNMR studies provided invaluable atomic-level insights into the transmembrane domain of the SARS-CoV-2 E protein, revealing a pentameric channel architecture and suggesting mechanisms of ion conductance and pH sensitivity.^3^ Our SEC-MALS data now provide direct, quantitative evidence of the E protein’s intrinsic ability to form a stable pentameric assembly in both detergent micelles and MSP nanodiscs, demonstrating that this oligomeric state is maintained across different membrane-mimicking environments.

The integration of structural and computational approaches in our study reveals important insights into the E protein’s conformational dynamics. NSEM and cryo-EM visualizations confirm the pentameric organization within nanodiscs, while MD simulations provide atomic-level insights into the dynamic nature of the C-terminal tail. Notably, our simulations reveal a striking environment-dependent behavior: the C-terminal tail is highly flexible when the E protein is embedded in an MSP nanodisc, but becomes significantly more stable and forms contacts with the membrane surface in a lipid bilayer. This finding suggests that the C-terminal domain plays an active role in membrane recognition and interaction, which may be crucial for the E protein’s functions in viral assembly and host cell interactions. The observed membrane thinning near the E protein pentamer, accompanied by asymmetric lipid bilayer deformation, provides a structural basis for understanding how the E protein contributes to membrane curvature during viral budding. These E protein–membrane interactions could be further modulated by post-translational modifications such as palmitoylation and glycosylation, as previously described, opening avenues for investigating how these modifications regulate E protein function.

The dynamic nature of the E protein’s C-terminal region, revealed through both cryo-EM and MD simulations, suggests the potential biological relevance of conformational flexibility in viroporin function. This intrinsic flexibility likely plays important functional roles in the protein’s interactions with host cell components, and may facilitate the E protein’s multiple functions during viral assembly and pathogenesis. The convergence of experimental and computational observations regarding C-terminal dynamics strengthens confidence in our structural model and provides a foundation for future investigations into how this flexibility is regulated in different cellular contexts.

Our successful reconstitution of the full-length E protein pentamer in MSP nanodiscs establishes a valuable experimental platform for future functional studies. The nanodisc system provides a well-defined, native-like membrane environment that can be systematically modified to investigate lipid-specific interactions, the effects of post-translational modifications, and the binding of potential therapeutic compounds. Future studies employing complementary approaches such as hydrogen–deuterium exchange mass spectrometry, advanced cryo-EM techniques with improved particle classification algorithms, or larger membrane-mimetic systems could further refine our understanding of the E protein’s structure–function relationships. Additionally, investigating the E protein in complex with other viral structural proteins, particularly the M protein, will be crucial for understanding the molecular mechanisms of coronavirus assembly and identifying potential therapeutic targets.

## Supplementary Material



## Notes

Additional experimental details, molecular dynamics simulation parameters, analysis, MALDI-TOF mass spectra, and supplemental SDS-PAGE figures (PDF)
