# Supplementary material for: Pentameric Architecture of the SARS-CoV‑2 Envelope Protein Revealed by SEC-MALS, Cryo-EM, and Molecular Dynamics
Source: J Phys Chem B. 2026 Mar 12;130(18):4770–9. doi: 10.1021/acs.jpcb.5c08730 (PMC13158902; doi:10.1021/acs.jpcb.5c08730)
Supplement: Supplementary file 1 [file jp5c08730_si_001.pdf]

Supporting information

Pentameric Architecture of the SARS-CoV-2 Envelope Protein Revealed by SEC-MALS, Cryo-EM, and Molecular Dynamics

Zi-Wen Weng,[1,2] Danny Farhat[3], Jyh-Yeuan Lee[3], Shang-Te Danny Hsu[1,2,3,4,*]

1. Institute of Biological Chemistry, Academia Sinica, 128 Academia Road, Sec. 2, Nankang, Taipei 11529, Taiwan
2. Institute of Biochemical Sciences, National Taiwan University, 1 Roosevelt Road, Sec. 4, Daan, Taipei 10617, Taiwan
3. Department of Biochemistry, Microbiology, and Immunology, Faculty of Medicine, University of Ottawa, Ottawa, ON K1H 8M5, Canada
4. International Institute for Sustainability with Knotted Chiral Meta Matter (WPI-SKCM^2), Hiroshima University, 1-3-1 Kagamiyama, Higashi-Hiroshima, Hiroshima 739-8526, Japan

* corresponding author: sthsu@gate.sinica.edu.tw

This file contains

Figures S1-4

Tables S1-2

Figure S1. Complete amino acid sequence of the synthesized SARS-CoV-2 Envelope (E) protein.

The sequence includes an N-terminal His-tag, highlighted in bold.

HHHHHHHHEN LYFQSFVSEE TGTLIVNSVL LFLAFVVFLL VTLAILTALR LCAYCCNIVN VSLVKPSFYV YSRVKNLNSS RVPDLLV

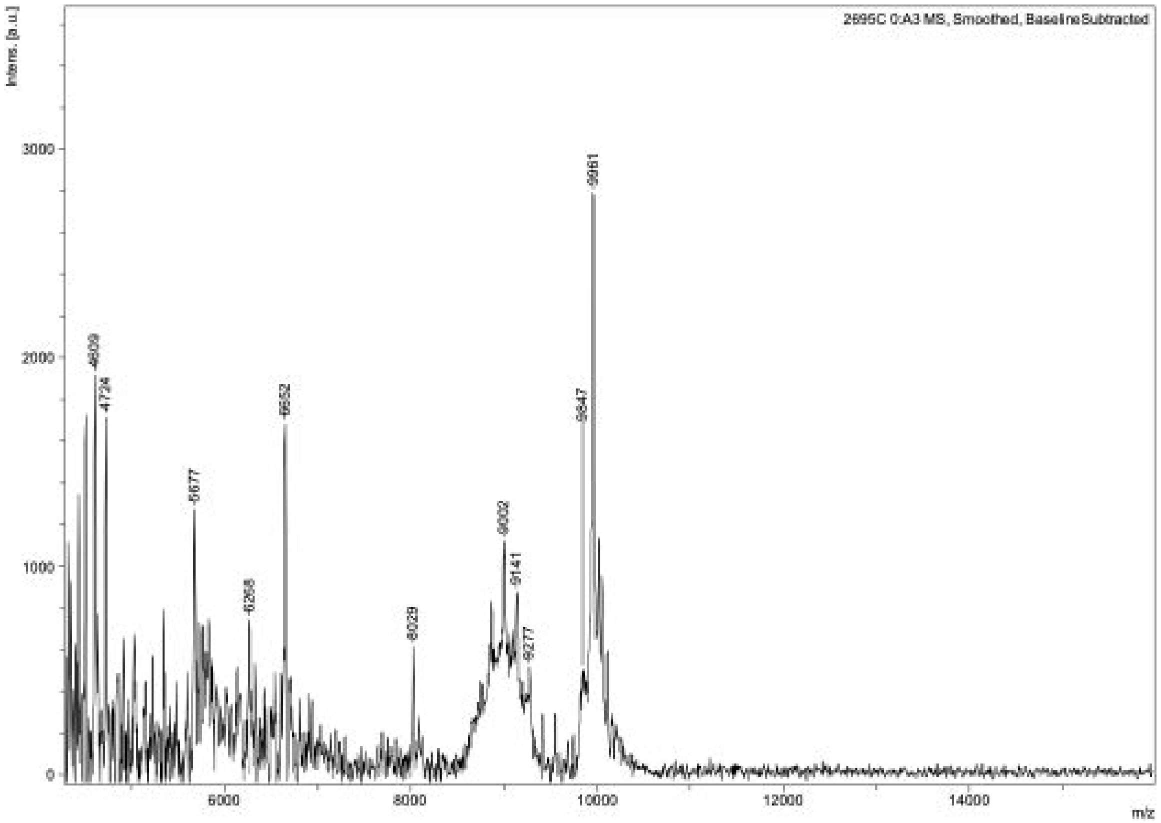

Figure S2. MALDI-TOF data of full-length synthetic His tag-E protein

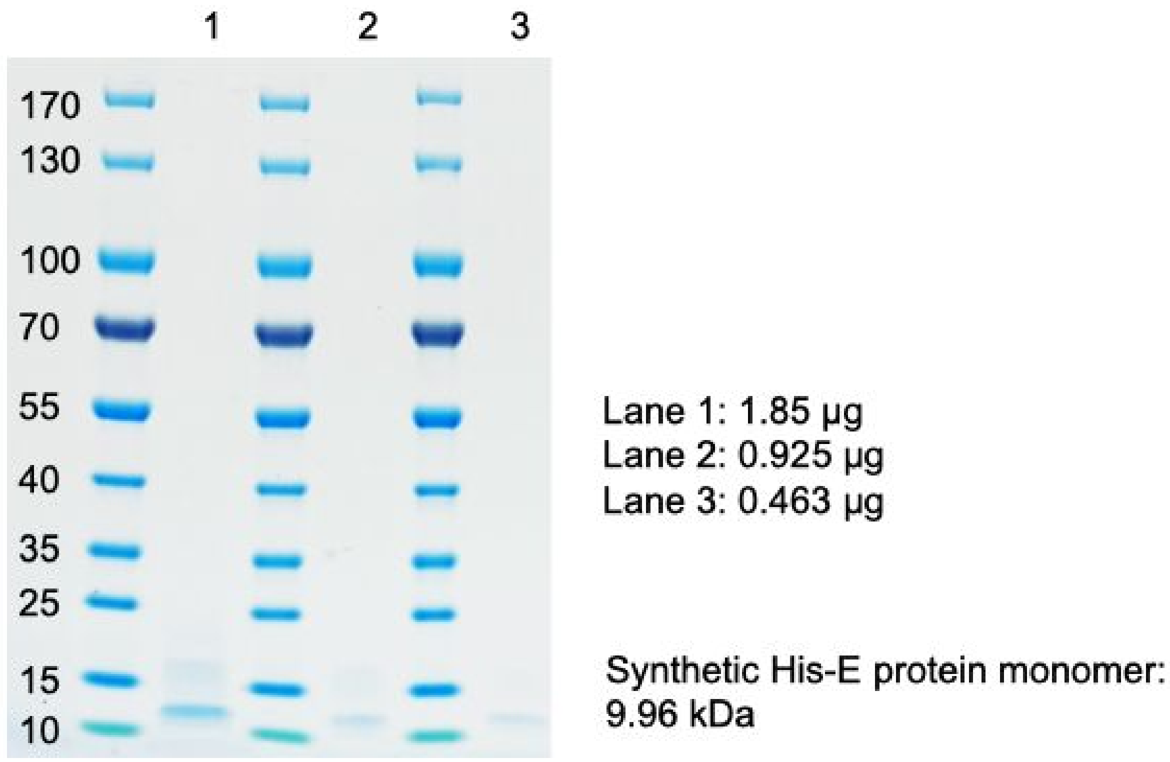

Figure S3. High-purity assessment of the FPLC-purified full-length SARS-CoV-2 E protein by SDS-PAGE. The FPLC-purified E protein was subjected to a two-fold serial dilution and resolved on an SDS-PAGE gel, followed by Coomassie Brilliant Blue (CBB) staining. Lanes 1-3 represent the serially diluted protein sample, with the first lane containing approximately 1.85 µg. The gel clearly demonstrates a single, highly distinct, and concentration-dependent band corresponding to the monomeric E protein (~10 kDa). Crucially, the complete absence of lower-molecular-weight bands confirms the successful elimination of SPPS-related truncation products. A trace amount of a higher-molecular-weight signal (between 15 and 25 kDa) is consistent with an SDS-resistant dimer, a common characteristic of highly hydrophobic integral membrane proteins. This result validates the structural homogeneity and high purity of the final sample utilized for all downstream structural characterizations.

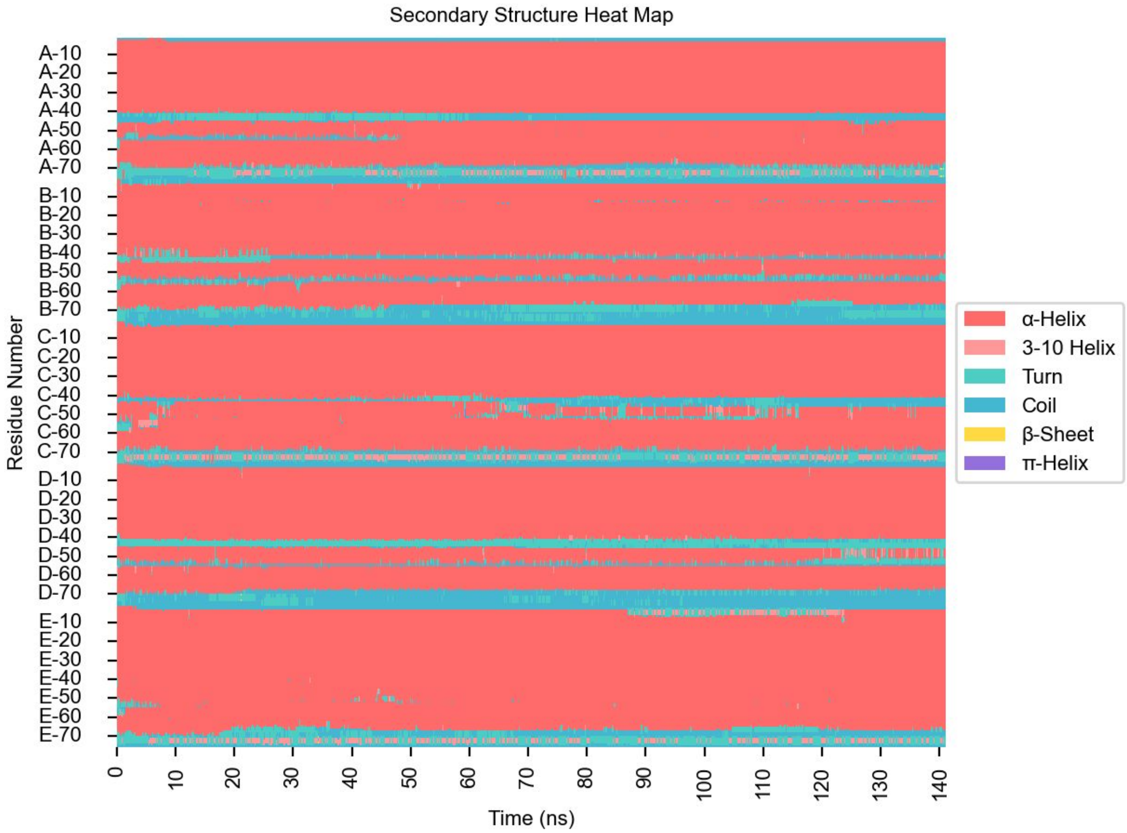

Figure S4. Secondary structure content of the E protein pentamer as a function of time

This heat map illustrates the temporal evolution of secondary structure elements for each residue in the pentameric assembly of the E protein throughout the molecular dynamics simulation. The x-axis represents the simulation time (in ns), while the y-axis corresponds to the residue number, which includes the concatenated sequences of all five monomers forming the pentamer. Different colors denote distinct secondary structure types, as indicated in the legend: α-helix (red), 3_{10} helix (orange), turn (green), coil (blue), β-sheet (cyan), and π-helix (purple). The predominance of red regions indicates that most residues maintain an α-helical conformation during the simulation, with occasional transitions to other secondary structures. This analysis provides insight into the structural stability and dynamics of the pentameric protein under the simulated conditions.

Table S1. Secondary structure composition of the E protein derived from CD spectroscopy.

Helix	Sheet	Turn	Other
55 %	3 %	10 %	32 %

Table S2. The system dimensions of the E protein in the membrane-mimicking MD simulations.

Protein	E-MSP1D1Δ5	E-Membrane
System dimensions (x, y, z; Å)	131, 145, 125	125, 125, 98
POPC	63	259
POPE	21	85
POPS	21	85
Sodium	57	145
Chloride	70	68
Water	32267	25328
Total Atoms	225830	138397
